# Prodigy Making

**Published:** 1869-04

**Authors:** B. H. Pratt


### Prodigy Making.

BY B. H. PRATT, A. M.

While the growth of the Republic witnesses proper and prudent growths in many directions, both within the sphere of the physical and intellectual—we are sorry we cannot add, the moral—it is also compelled to lament enervating processes, as foolish as they are harmful, and as unjust as they are unwise.

This is more to be wondered at in the matter of which this paper treats,—early education—inasmuch as we are apt to plume ourselves particularly as progressionists in this role—improvers upon the world's old way of doing things—disregarders of time-worn maxims—levelers of mountain prejudices—disinfectors of musty conceits—iconoclasts, in a general way—of matters dogmatical, but master-workers in cosmopolitan amelioration.

The writer does not decry the spirit that underlies this pride and boast. On the contrary, as a defender of our institutions, sublime as they are in most particulars, no hat will fly higher—no huzza echoes wider—no zeal gleam more radiantly than his; but while art, in much, is the inferior of nature—while brute force is not always power—while push is not always progress—he must lift his voice against a system, ruinous alike to the body and the head and the heart of the "coming man."

For the sake of illustration, note the modern system of juvenile trainers—parental prodigy perfectionists. Juvenis, born of reasonably intelligent and highly cultivated parents—as men count intelligence and culture—who feel the ponderous responsibility attaching to the office of rearing a possible president, probable congressman, certain sure great man at least—gets comfortably through the perils of croup, back stair somersaults and first breeches. Several alphabetical and numerical spectres may have appeared in the mean time, but by no means ghastly, when, at three or four years of age, he is seized, *a tergo*, and thrust into the very midst of that juvenile horror of horrors, a school room. To him "the Master" is a fiend incarnate, armed with powers plenipotentiary for his special torment—every boy bigger than himself an imp, ready to pounce upon and rend him—every lesson an instrument of torture, hourly pointed for his special prod. Youth is not to be blamed for its timidity, nor yet for its lack of intellectual appreciation. Juvenis sees no practicability in the parental scheme further than the attainment of his own absolute misery. True, time blunts many of the keen points of scholastic pricks, and he perhaps becomes reconciled to his martyrdom.

Nor is the atmosphere of a school room remarkable for its purity. The initiated know full well the miasmatic effluvia which emanate from decomposing apple cores—rotting fragments of fruit clandestinely introduced—exhalations from the persons of the notoriously unclean—to say nothing of hourly accumulating volumes of carbonic acid gas, the natural result of multiplied respirations, however well the theory of ventilation may have been practicalized. The delicate organizations of tender years are not capable of counteracting these effects, and the school-room must be held responsible for the hosts of pale faces, pinched features and puny forms that meet us daily at every turn.

The spirit of youth—the keener instincts of its moral nature—its entirety of soul, so to speak—departs with the loss of physical vigor. A soul's malformation, developing hideously day by day, under your very eye, is one of the visions ever attendant upon the life of preacher and teacher alike; though to the latter more terribly shocking, in that he sees the cause and remedy, but is powerless to remove the one or apply the other. So hatreds grow that will prove life-long—hatred of school—hatred of books—hatred of teacher—hatred of mankind—aye, hatred of parents—for the manifold effects of which we refer you to our criminal records, our prison catalogues, and the hoary heads gone, and now going, down with sorrow to the grave. Thus, instead of the beautiful development of love, and the lovely development of the beautiful in childhood, Juvenis, is at ten, a dullard—an intellectual dwarf—a puny wretch, physically, mentally and morally—a misanthrope—a blighted soul. There be exceptions, but this is the rule.

Let any parent, whom this shoe fits, call to mind his own school experience. Does he not remember—if he was so unfortunate as to be one of these early grafts upon the tree of knowledge—how, at the age of ten or eleven, some farmer's lad, of ruddy cheek and stalwart limb entered his school? How dumb you thought him then; but ah, how studious! And what strides he made from the lowest to the highest of the branches there taught, mastering his studies at every step, thoroughly in earnest, and with physique enough to back any amount of brain work his indomitable will demanded. How he fairly devoured learning, leaving gentle Juvenis far in his wake. Arouse your memories of academic or collegiate days, and confess how many of your fellow workers, whose initial ignorance raised the laugh in class, have passed you in the race of life, and whose names stand above your wildest ambition to day.

Previous to the age of eight—aye, even ten—the body needs more nurture than the mind. From ten to fifteen you may work both equally hard, adjusting the balance as nicely as you may. From fifteen upward, if you have not broken faith with the body, much study will *not* be a weariness to the flesh, nor *can* it be. Who are our broken down—health hunting—cough doctoring—wheezing—voyage taking—leave asking ministers? Victims of prodigy making parents. Who are our hard muscled—jovial souled bible pounders and pulpit bangers—our deep chested—deep thinking—fear ignoring clergymen? Farmers' boys—mechanics' boys—who had to work *before* they could afford to learn from books.

But bodily harm is perhaps the least evil engendered by prodigy making. The intellect never grows in the weak body beyond a certain limit. To that it may leap like a flash, but the fuel is scarce and it soon burns low. Hence our surprise at the lack of intellectual *growth* in many a man possessed of wondrous genius in the main. Standing still, the world passes by him, and warm admirers find their bud of much promise and prospective beauty fading ere it really bloomed.

But most of all does the *moral* nature of boy and man suffer by this sacrifice of the physical to the intellectual; and the saddest of all sad sights is this picture of the sinking from view of the better qualities of the juvenile heart, and the rising into view of all the baser passions of man's evil nature. The imprisoned youth, impatient of restraint—galled by the consciousness of injustice being done him—tempted, too, by hours upon hours of stolen idleness—plots; plots, perhaps feebly, as men rate plotting—plots, perhaps because to plot is the result of coerced quietude—plots because he can't help plotting. Plots what?—Good? My dear friend, you who couldn't be induced to believe aught ill of that cherubic youth of yours—that sweet saint in little breeches, who comes to you with all his pretty feathers waving nobly and all his nasty quills poked out of sight—that innocent darling is a devil in disguise and *you* are the only one that never will see it, until it glares upon you from the prisoners' box, or from between the bars of the felons cell, whither your mistaken zeal may in after years have driven him. Alas, for the blindness of parents to the sins of their own! Alack, for the keen sightedness of the same for the errors in everybody else's darlings! My dear Sir, and my honored Madam, I'll tell you a secret; *your boy is a devil.* It's only grace that makes saints, but there's not grace enough for all the big folks, and the little ones, God help them, poor things, get none, and would rather not have it any how. Now the only way given under heaven among men, whereby a boy, pattern exact of the old Adam, can be kept free from the assaults of himself, is by keeping his body active, and quenching thought until thought has got a balance wheel of physique to regulate it.

But in an article brief as this must needs be —brief indeed, were the editor kind enough to grant me the whole of the present volume for this important subject—scarcely a tithe of the evil effects of too early mental application can be portrayed. Enough has been said, however, to arouse thought upon the subject, and we will pass briefly to an investigation of the causes of the system we so sincerely condemn.

It is a failing with most parents to see evidences of wondrous smartness in their offspring upon ridiculously feeble grounds. Each infantile smirk, trick, lisp, is magnified into portentous meaning. No child of natural abilities ever lived, probably, of which its parents or relatives have not sometime said "it's too smart to live," or "I fear we'll never be able to raise that child." Here is the origin of the whole difficulty. Does baby in a purely natural way evince a fondness for any particular toy? its idiosyncrasy is at once determined. Does he delight in any particular practice above all others? immediately the horoscope is applied and his destiny revealed. But mind you, it's always a grand—a magnificent destiny. Does he carefully scan his own hands, feet, legs, toes? he will become an eminent physiologist. Does he take to blocks? an architiect. Does he pick pebbles, and exhibit a fondness for mud pies? a geologist. Does he gather bugs, and prison flies, and grab toads? a naturalist in embryo. Does he, in imitation of his seniors seize a book and gabble over it? he will one day be the poet laureate. Nonsense! Every youngster does most, if not all of these, and has done so for centuries, while nine-tenths of them, grown up, have shown no genius at all. But prodigies they are, in parental eyes, and so they shall be; and just for fear that they may not become developed with that celerity commensurate with a parent's impatience, they are forced into intellectual hot-beds.

This mania to "show off" children—this ambition to be known as the parent of a prodigy—has killed more bodies than the plague has—dwarfed more minds than alcohol has—damned more souls than the Inquisition has. If parents *could* only look upon their children as they are—prodigies, *if* such—mediocres, *if* such—what a world of anxiety and suffering might be avoided; how much higher the standard of the nation's physical, intellectual and moral culture would rise.

The easiest to suggest, yet the most difficult to apply, is the radical cure for this self imposed national calamity. It is easy to say, let your boys alone for seven years, so far as intellect is concerned. But must we *ignore* the juvenile brain? By no means. The normal brain, be it encased in what encephalon it may, is accretive. But let there be no pushing. Oral lessons, so long as interesting, and not one second longer, may be taught—precepts instilled at the fitting moment, when they can be masked under an attractive exterior. But the great business that mother nature has for her children, is play. Play! the great relaxer–Play! the great re-creator—thank God for Play! It is the goal that makes the shop race—the office race—the life race endurable. We work and toil and tire—we sweat and suffer and slave—that we may play; if not sooner, then later. Manhood strives to accumulate wealth that it may buy play for age; and O, wretched he, who, by reason of his long work has no desire for play.

Let the boy play—out doors, in doors, everywhere—play and sleep beautifully alternating, until play merges into work, and work into study, and sleep blesses the trinity.

Children that are always pretty to see by reason of virgin dimity and spotless cuticle, are poor, miserable little wretches in the eyes of the practically philanthropic. Only weak parents fear the results of play. Miserable are ye, whom a garment rent beyond recall disturbs, and from whom a smutty face, and hands right well besmeared, evoke parental wrath and birchen injustice.

